# Chicago Pathological Society, Meeting of August 13

**Published:** 1883-10

**Authors:** J. H. Tebbetts

**Affiliations:** Secretary


### ARTICLE IX.

CHICAGO PATHOLOGICAL SOCIETY. Regular meeting August 13, 1883.

The society was called to order by the President, Dr. Angear. The minutes of the last meeting were read and approved.

Dr. Fannie Dickinson was proposed for membership by Drs. J. E. Haynes and D. R. Brower.

The society then listened to the reading of a paper by Dr. Brower, entitled "Notes of a Fatal Case of Chorea, Its Pathology and Treatment."

This paper, which the author stated would subsequently be published in THE CHICAGO MEDICAL JOURNAL AND EXAMINER, gave a full and detailed report of a very interesting case of chorea, occurring in a female, with a fatal termination.

Dr. Brower, after reading the notes of the case, remarked his interest in the subject of heart lesions without murmurs. The case of Trousseau was the only one he knew of where lesions were present but no murmurs were observed.

The paper being before the Society for discussion, Dr. Lyman stated that in some cases a change of position would develop a murmur when not noticed in a sitting posture. Auscultation after fast walking or running, and sometimes in the recumbent position, would alone reveal a murmur otherwise undetected.

Dr. Angear remarked that as the murmur would depend upon the obstruction to the circulation or the roughness of the valves, if the disease had not caused this roughness or obstruction to any considerable extent, the sounds would be too delicate to be detected without special instruments. Temporary heart difficulty might cause embolism, which produced its effects after the heart had regained its tone. The point that chorea was a paresis of the nerves was a disputed one; this case supported the view, for, the brain being affected, chorea appears instead of paralysis or softening.

Dr. Lyman thought that the lesions could be predicted if any were found. Most cases recover. It was not common to find embolic obstruction as in above case; sclerotic patches sometimes appear in choreiform cases ; this case showed the characteristic lesion.

Dr. Brower thought that in recently reported cases lesions had always been found, generally in the left corpus striatum. In cases reported some time ago, there was not the care and pains taken in examination. Sixty-five per cent. of cases show heart trouble.

Dr. Lyman stated that if obstruction existed in the heart, and emboli were detached and carried to the brain, the right side of the brain would be liable to be affected. He then inquired what treatment seemed most valuable iu the case reported.

Dr. Brower, in reply, stated that the general treatment was unsatisfactory ; that morphia, in $\frac{1}{2}$ gr. doses, would quiet the patient somewhat. He used chloral in 40-grain doses in vaginal injections. This had no effect, as also drachm doses of potassium bromide and 1-30 gr. doses of hyoscyamine. Chloroform was used once only as it oppressed her breathing.

In order to cease movement, she must sleep under morphia, in $\frac{1}{2}$ gr. doses, which had less effect than $\frac{1}{4}$ gr. to the ordinary patient. After a profound sleep, she had more movement than before. In ordinary cases of chorea in children, Dr. Brower gives Fowler's solution prepared without the lavender, in four drop doses, hypodermically, four times a day.

Children bear arsenic wonderfully well ; in one case of a child, gave eight drops three times a day, before the paroxysms were controlled. He had never seen any bad effects from its use. This statement was endorsed by Dr. Lyman. Strychnia he considered of less value. To the general treatment he always pays careful attention ; uses port wine in large doses generally with citrate of iron ; three or four wineglassfuls a day will generally produce sleep better than an anodyne. The iron should be given when indicated, and it generally is—preferably the milder preparations.

The use of the battery is difficult with children. Employs ether spray to spine with good effect.

The society then listened to a verbal report of a case of infantile tetanus, by Dr. E. P. Murdock. The age of the patient was five days. Dr. Murdock was summoned in the afternoon, and arrived at 8 P.M. Found child in a convulsion. The spasm was a most violent one; the features cyanosed and emaciated, and the whole frame greatly shrunken in twenty-four hours, as stated by parents. The spasm soon relaxing, breathing became easier, but at touch the child again convulsed. A touch upon the chair upon which the child's bed was made would cause a convulsion; the abdominal muscles were rigid, and an irregular reddish spot appeared about the umbilicus, of the color of eczema rubrum; the bowels had been regular; the temperature 100°; deglutition difficult. Gave tincture physostigma, chloral, and potassium bromide. Morning found the child weaker; prostration extreme; diaphoresis and diuresis profuse; child could not swallow the medicine. Called Dr. Angear in consultation, who advised the same medication administered per rectum. In the afternoon, the temperature was 108.5°, and death occurred.

Among the interesting features of the case were, 1. The spasm was excited by fanning. Justifiable force could not bend the arm or move the jaw, while complete relaxation occurred after the spasm. 2. The peculiar redness about the umbilicus. 3. The high temperature before death, 108.5°. He had never observed a temperature so high.

Dr. Brower then mentioned two cases of tetanus in the practice of Dr. Hosmer Johnson in one year, cured by large doses of quinine. Cases of "toy-pistol" tetanus had recovered under full doses of quinine. Dr. Jones, of New Orleans, reports recoveries from tetanus by use of quinine.

Dr. Lyman would ask if Dr. Murdock had noticed the condition of the bones of the cranium? Some one who had had considerable experience noticed a displacement of the cranial bones, the parietal overlapping the occipital. By replacing these bones, he had relieved a majority of cases of tetanus. This was the only allusion of which he had ever heard to this condition. It would be well to keep it in mind.

Dr. Murdock had noticed depression of the fontanelle, but not overlapping of the bones. The residence and surroundings, locality, etc., of the patient were of the better variety; no indication of foul air or filth of any kind.

The Society then listened to the final paper of the evening, by

Dr. Walker, upon the operation of "Lithotomy," end report of case in a child seven years of age. It was the left lateral operation, with a very favorable result.

The operation in boys, the author stated, presented several difficulties—

1. Owing to shortness of the perineum, the first incision is very near the scrotum.

2. The bladder lies high, rather above the pelvis in the abdominal cavity; so that the incision, if not upward, must be at least straight ahead—not downward.

3. If the incision should prove too contracted, the membranous urethra may be torn across with the finger, and push the bladder backward, the least diastrous result of which would be a traumatic stricture.

The paper being before the society for discussion, Dr. Lagorio remarked a case of a child, three years of age, as operated on. The child played about the floor on the third day, and was entirely recovered in three weeks.

Dr. Angear in 1879 saw Pollock, of London, operate on a four year old boy, using a straight staff, a pocket scalpel and small dressing forceps, exposing and removing a stone in as many moments, as he (the narrator) was using in description. The fuss was less than in extraction of a tooth.

An application for membership was then made by letter from Dr. Erichsen, Detroit, Mich., but as by constitution all members shall be residents of Chicago, the Secretary was requested by motion to so inform the gentleman.

Among those present were Drs. Angear, Brower, Beecher, Dobbin, Harper, Hayner, Lagorio, Lyman, Murdock, Newton, Patton, Tebbetts, Walker, and four visitors.

The Society on motion adjourned.

J. H. TEBBETTS, Secretary.

